# ZNF185 is a p63 target gene critical for epidermal differentiation and squamous cell carcinoma development

**DOI:** 10.1038/s41388-018-0509-4

**Published:** 2018-10-18

**Authors:** Artem Smirnov, Anna Maria Lena, Angela Cappello, Emanuele Panatta, Lucia Anemona, Simone Bischetti, Margherita Annicchiarico-Petruzzelli, Alessandro Mauriello, Gerry Melino, Eleonora Candi

**Affiliations:** 1 Department of Experimental Medicine and Surgery, University of Rome “Tor Vergata”, via Montpellier, 1, 00133 Rome, Italy; 2 Istituto Dermopatico dell’Immacolata-IRCCS, via Monti di Creta 104, 00163 Rome, Italy; 3 MRC-Toxicology Unit, Hodgkin Building, University of Cambridge, Hodgkin Building, Lancaster Road, Leicester, LE1 9HN, UK

**Keywords:** Oral cancer, Adherens junctions, Differentiation

## Abstract

Development and maintenance of healthy stratified epithelia require the coordination of complex transcriptional programmes. The transcription factor p63, a member of the p53 family, plays a crucial role in epithelial development and homeostasis. Analysis of the p63-dependent transcriptome indicated that one important aspect of p63 functions in epithelial development is the regulation of cell–cell and cell–matrix adhesion programmes. However, limited knowledge exists on the relevant cell–cell adhesion molecules involved in physiological epithelial formation. Similarly, limited data are available to understand if deregulation of the cell–cell adhesion programme is important in tumour formation. Here, using the epidermis as an experimental model with the RNA sequencing approach, we identify a novel p63-regulated gene induced during differentiation, ZNF185. ZNF185 is an actin-cytoskeleton-associated Lin-l 1, Isl-1 and Mec-3 (LIM) domain-containing protein, whose function is poorly known. We found that p63 binds to a specific enhancer region, promoting its expression to sustain epithelial differentiation. ZNF185 silencing strongly impaired keratinocyte differentiation according to gene array analysis. ZNF185 is detected at the cell–cell periphery where it physically interacts with E-cadherin, indicating that it is important to maintain epithelial integrity beyond its pro-differentiation role. Interestingly, poorly differentiated, including head and neck, cervical and oesophageal, squamous cell carcinomas display loss of ZNF185 expression. Together, these studies reinforce that p63 is a crucial gene for maintaining epithelial tissue integrity and support the deregulation of the cell-cell adhesion programme,which plays a critical role in carcinoma development.

## Introduction

A complex transcriptional programme is required for epithelial formation and stratification [1–14]. Epithelial cells undergo a multistage differentiation programme that starts with the migration of the proliferating keratinocytes towards the upper outermost layers. The differentiation programme ends with the formation of a layer of dead keratinized cells, named the cornified layer. Here, the plasma membrane has been replaced by a specialized structure, the cornified cell envelope; together with adhesion complexes, the cornified cell envelope is responsible for structural integrity, elasticity and barrier function of the epidermis [14]. Cell-cell adhesion structures, including adherens junctions, tight junctions and desmosomes, are the most important cellular structures to guarantee a functional epithelium; they promote interaction among cells and allow for stratification and differentiation [15–18]. Indeed, cytoskeleton rearrangements and cell adhesion are crucial for organizing cells into a three-dimensional tissue during normal development, providing integrity and barrier function of the skin, and contributing to wound healing and tumorigenesis [19–24].

p63, a transcription factor member of the tumour-suppressor p53 family, has a very important role in epithelial morphogenesis and stratification [4]. Mice lacking p63, or the amino-deleted isoform ΔNp63, fail to develop epidermis, simple epithelia, skin appendages, and limbs, indicating that p63 is the master gene that initiates the specific programme for epidermal cell determination and stratification starting from the single ectoderm layer [5, 6]. Multiple gene groups, including PERP, β-catenin, integrins α6 and β4, laminins, fibronectin, several collagens and collagen receptors, involved in many aspects of cell adhesion, have been described to be regulated by modulation of p63 levels [7–13]. Nevertheless, only a limited number, including PERP, laminins and integrins, have been demonstrated as bona fide p63-target genes with a specific in vivo function [7, 9]. Thus, the underlying mechanisms by which p63 engages specific pathways to determine epithelial stratification, maintain tissue integrity, and alter the normal architecture and homeostatic mechanisms of stratified epithelia in carcinoma development remain elusive.

Although several investigations have highlighted the crucial components of the keratinocyte differentiation process, further details of the underlined molecular events are required. To this end, we set out to perform an RNA sequencing experiment of in vitro differentiating normal human keratinocytes. In particular, we focused on genes upregulated during the differentiation process and on the relationship with the above-mentioned p63. Here, we demonstrated that ZNF185 is an important player in the p63-dependent transcriptional programme directed to the development of stratified epithelia. Genome-wide analysis, ChIP-seq data sets, gene-specific ChIP experiments and RT-qPCRs revealed that p63 uses a specific active enhancer sequence, which is approximately 20 kb up-stream TSS, to transcribe ZNF185. Depletion of ZNF185 in keratinocytes strongly reduced calcium-induced gene expression. ZNF185 is restricted to the upper differentiated layers of the epidermis at the cell–cell periphery, and it interacts with E-cadherin, a component of adherens junctions. Interestingly, E-cadherin and ZNF185 expression levels are strongly downregulated in poorly differentiated, including head and neck, cervical and oesophageal, squamous cell carcinomas. These results provide evidence that ZNF185 is a key component of the p63-dependent transcriptional programme for stratified epithelial development and tissue homeostasis, as well as that ZNF185 deregulation, beyond deregulating cell adhesion in general, is also relevant for carcinoma formation of the stratified epithelia, including skin, oesophagus and cervix. ZNF185 could be considered a potential specific biomarker for SCC diagnosis and prognosis.

## Results

### RNA sequencing identifies ZNF185 as a highly expressed gene during keratinocyte differentiation

**Fig. 1:**
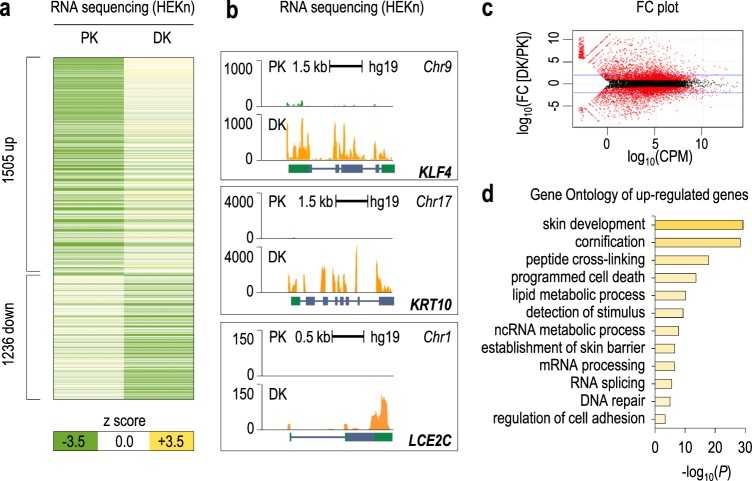
RNA sequencing in keratinocytes reveals new genes involved in differentiation. **a** Heat map representing *z* scores of gene expression values determined by RNA-seq in proliferating (PK) and differentiated (DK) keratinocytes HEKn. *P* < 0.05; abs(log*FC*) > 2. **b** Genomic loci of *KLF4*, *KRT10*, and *LCE2C* and their enrichment scores obtained by RNA-seq in PK and DK HEKn. **c** Expression ratio scores of each gene in differentiated/proliferating samples. FC plot is generated using edgeR Bioconductor, which shows the distribution of modulated genes from (**a**) (logFC) as a function of their expression (logCPM) obtained from the RNA-seq in PK and DK HEKn. **d** GO terms for enriched genes in HEKn differentiation obtained from RNA-seq

**Fig. 2:**
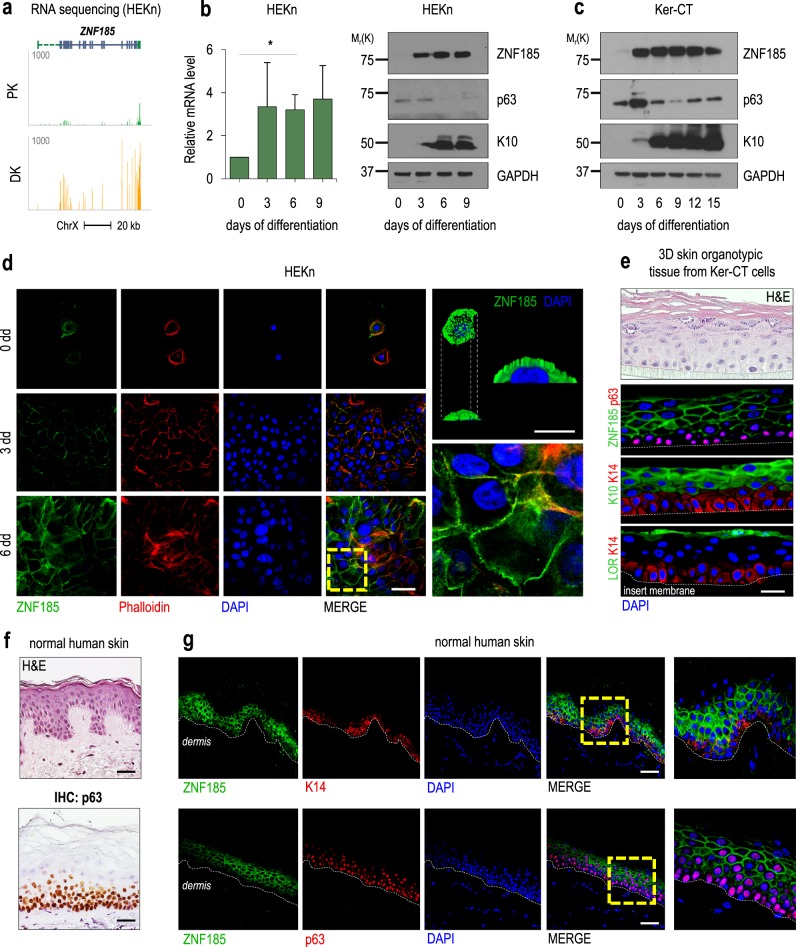
ZNF185 is upregulated during keratinocyte differentiation and in human epidermis. **a** RNA expression of *ZNF185* in PK and DK HEKn analysed by RNA-seq. **b** RT**-**qPCR analysis of the *ZNF185* mRNA level in HEKn that had been differentiated for 0, 3, 6, and 9 days. *P < 0.05. Western blot showing the expression levels of ZNF185, p63, and K10 in HEKn differentiated for 0, 3, 6, and 9 days. **c** Western blot showing the expression levels of ZNF185, p63, and K10 in Ker-CT cells differentiated for 0, 3, 6, 9, 12, and 15 days. **d** Immunofluorescence analysis of ZNF185 expression in HEKn differentiated for 0, 3, or 6 days. Scale bar: 50 µm. Right panel: 3D rendered confocal imaging of HEKn using alpha-blending algorithm. Scale bar: 20 µm. **e** Immunofluorescence analysis of ZNF185, K10, Lor, and p63 expression in 3D organotypic skin tissue from Ker-CT cells. Keratin 14 was used as a marker of the basal layer. Scale bar: 50 µm. Upper panel: H&E staining of the 3D tissue. **f** H&E and IHC for p63 staining of normal human skin. **g** Immunofluorescence analysis of ZNF185 and p63 expression in human epidermis. Keratin 14 was used as a marker of the basal layer. Scale bar: 100 µm

Calcium-induced differentiation of keratinocytes is an important step in the normal development of the human epidermis. We performed RNA sequencing of proliferative (PK) and 6-day differentiated (DK) keratinocytes to investigate novel genes involved in keratinocyte differentiation (Fig. 1). Expression of protein-coding genes with an average RPKM (reads per kilobase per million mapped reads) of >1 was scored as a ratio of differentiated to proliferating transcripts. We identified 1505 upregulated and 1236 downregulated transcripts (abs(log(FC)) > 2) (Fig. 1a–c, Supplementary Table S2). Gene Ontology (GO) analysis of the most enriched transcripts in differentiating conditions showed significant distribution among skin development groups, including cornification, peptide cross-linking, and cornification and cell adhesion (Fig. 1d, Supplementary Table S5). As a positive control, we checked the RNA expression of some genes known to increase during differentiation, e.g., *KLF4*, *KRT10*, and *LCE2C* (Fig. 1b). Interestingly, GO analysis revealed 149 unclassified genes among the most enriched transcripts in differentiating conditions, including ZNF185, a LIM-domain Zn-finger protein with unknown function (Fig. 2a). RNA sequencing data were further confirmed by analysing mRNA and protein levels of ZNF185 at 0, 3, 6, and 9 days after CaCl_2_ treatment (Fig. 2b). These data were also confirmed using a commercial cell line of immortalized human keratinocytes, Ker-CT, which is suitable for in vitro experiments (Fig. 2c). To study the subcellular localization of ZNF185, we performed immunofluorescence staining of proliferating and differentiating human primary (HEKn) keratinocytes (3 and 6 days). Confocal microscopy analysis confirmed that ZNF185 is up-regulated in differentiated keratinocytes and revealed the cell membrane localization of ZNF185 (Fig. 2d, Figure S1a). The staining at the cell membrane was also confirmed both in 3D skin organotypic tissue obtained using Ker-CT cells (Fig. 2e) and in human skin (Fig. 2f, g). In all experimental models tested, ZNF185 was detected in the differentiation layers at the cell–cell boundary. These data demonstrated that ZNF185 is a membrane- and cytoskeleton-associated protein and that it is strongly associated with epidermal differentiation.

### ZNF185 is a p63 target gene

**Fig. 3:**
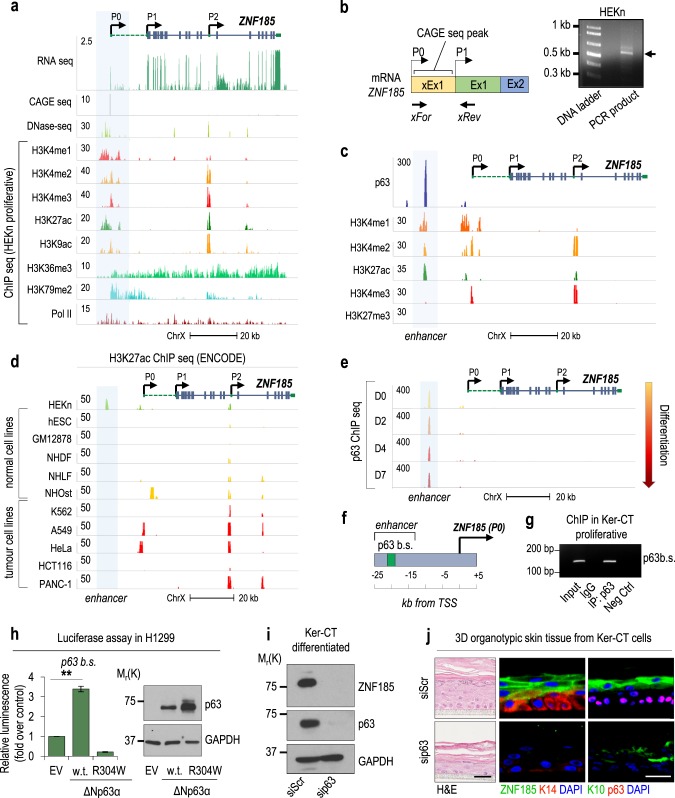
p63 regulates ZNF185 expression in keratinocytes. **a** Genomic locus of *ZNF185* analysed for RNA-seq, CAGE-seq, DNase-seq, H3K4me1, H3K4me2, H3K4me3, H3K27ac, H3K9ac, H3K36me3, H3K79me2, and Pol2 ChIP-seq signal tracks. Data presented were obtained from ENCODE portal. **b** Scheme and results of amplification of predicted xEx1 sequence within 5’-UTR of *ZNF185* mRNA from differentiated HEKn. The arrow indicates the PCR product. **c** The genomic locus of *ZNF185* showing the putative enhancer region with p63 (accession number: GSM1446927), H3K4me1, H3K4me2, H3K27ac, H3K4me3, and H3K27me3 ChIP-seq signals. ChIP-seq data for histone modifications were obtained from ENCODE portal. **d** Enhancer of *ZNF185*, analysed by H3K27ac ChIP-seq in different cell lines. Data presented were obtained from ENCODE portal. **e** Enhancer of *ZNF185* with enrichment of p63 ChIP-seq signal during keratinocyte differentiation (accession numbers: GSM1446927, GSM1446928, GSM1446929, and GSM1446930). **f** Identified p63 binding site (p63b.s.) within *ZNF185* enhancer region. **g** Amplification of specific DNA fragments after ChIP performed in proliferating Ker-CT cells using p63 antibody. **h** Luciferase activity assays in H1299 after transfection of pGL3-*ZNF185* enhancer and empty vector, ΔNp63α or ΔNp63α-R304W expression vectors. **P < 0.01, *n* = 4. Western blot analysis of cell lysates to confirm ΔNp63 overexpression. **i** Western blot analysis of ZNF185 and p63 levels in differentiated Ker-CT cells with p63 depletion. **j** Immunofluorescence analysis of ZNF185 in organotypic skin tissue based on Ker-CT cells with p63 depletion. K14 and p63 were used as markers of the basal layer, and K10 was used as a marker of the upper layers of epidermis. Scale bar: 50 µm

To investigate ZNF185 regulation at the transcription level, we studied the transcription factors implicated with and binding to the ZNF185 promoter region. Since this genomic region is poorly studied and the precise position of transcription start site (TSS) of *ZNF185* is not clear, we decided to study the *ZNF185* promoter. The structure of *ZNF185* gene (Fig. S1b) reveals that ZNF185 has two annotated (P1 and P2) and one predicted (P0) transcription start sites. Several transcript variants arising from P0 and P1 differ in their 5’-UTR but possess the same coding sequence. Analysis of RNA sequencing peaks revealed that 15 kb up-stream of annotated TSS of *ZNF185* (P1) there is an additional peak that corresponds to the TSS (P0) of several predicted isoforms of *ZNF185*. To better understand the *ZNF185* promoter structure and real TSS position, we performed analysis of CAGE sequencing (showing the 5’-cap associated region), DNase sequencing (showing the accessible regions of genomic DNA), and ChIP sequencing for H3K4me1, H3K4me2, H3K4me3, H3K27ac, H3K9ac, H3K36me3, H3K79me2, and Pol2 (markers of open chromatin and active promoter) performed in proliferative keratinocytes grown in standard conditions (Fig. 3a). Interestingly, none of the indicated open chromatin markers was present in the region of annotated TSS P1; meanwhile, predicted TSS P0 had high enrichment in indicated histone modifications, Pol II and unmethylated DNA (Fig. 3a). CAGE sequencing confirmed the presence of TSS precisely at P0. PCR performed on a template cDNA obtained from differentiated HEKn using the indicated primers (xFor, xRev; Fig. 3b) confirmed amplification of the sequence corresponding to the CAGE seq-peak. Sequencing of the PCR product confirmed the presence of a predicted part of the 5’-UTR in the mature RNA of *ZNF185* (Fig. S1c–d). Analysis of the sequence (circa 400 bp) did not reveal any Kozak sequence, indicating that it could be part of the 5’-UTR rather than the coding sequence (data not shown).

Since the analysis of the gene array performed on E18.5 WT or p63 null mice skin revealed a four-fold decrease in *Zfp185* gene expression in p63 null mice skin compared to the control [25], we evaluated whether p63 is bound to the *ZNF185* promoter region. Firstly, by analysis of publicly available RNA sequencing data from the skin biopsies using GTEx portal (Fig. S2a) and RNA sequencing data of proliferative and differentiated keratinocytes (Fig. S2b) from Fig. 1, we confirmed previously published data that show ΔNp63α as the major p63 isoform expressed in the human keratinocytes and human epidermis. We identified several putative binding sites for p53 family members and performed analysis of publicly accessible ChIP sequencing data; however, we did not observe any strong binding of p63 to *ZNF185* promoter. Interestingly, recently it has been shown that p63 acts as a co-activator binding enhancer regions rather than promoter regions [26]. Thus, analysis of ChIP sequencing for p63 in HEKn carried out by different groups [26–32] in the −50 kb/ + 50 kb region revealed the presence of a strong binding site approximately 20 kb up-stream of the *ZNF185* TSS (Fig. 3c, Figure S3a). Interestingly, we found that this genomic region is highly accessible, as indicated by ChIP sequencing for different histone modifications (ENCODE) showed high levels of H3K4me1, H3K4me2, and H3K27ac with low levels of H3K4me3 and H3K27me3 (Fig. 3c); furthermore, the H3K27ac marker increases during differentiation (Figure S3c). Additional analysis also showed that p63 binds this enhancer during in vitro-induced differentiation [30] and that this enhancer is keratinocyte-specific (Fig. 3d, e) and highly conserved (Figure S3b). Chromatin conformation capture in differentiated HEKn depleted for p63 showed that the enhancer/promoter interaction is only maintained in the presence of p63 (Figure S3d–e). p63 binding to the identified enhancer was confirmed by ChIP performed in proliferative Ker-CT cells using p63α specific antibody (Fig. 3f, g). Then, we subcloned the enhancer region up-stream luciferase reporter gene. We observed a three-fold increase in luciferase activity upon ΔNp63α over-expression. This activity was abrogated by using one of the DNA-binding domain mutants, ΔNp63α-R304W (Fig. 3h). Regulation of the expression of ZNF185 by ΔNp63α was also confirmed at the protein level (Fig. 3i, j). In fact, RNAi knockdown of p63 showed a strong reduction in ZNF185 compared to the control in vitro and in 3D organotypic skin. Altogether, our findings indicate that ZNF185 expression is regulated by p63 using a novel epithelial-specific enhancer.

### ZNF185 allows keratinocyte differentiation and interacts with E-cadherin

**Fig. 4:**
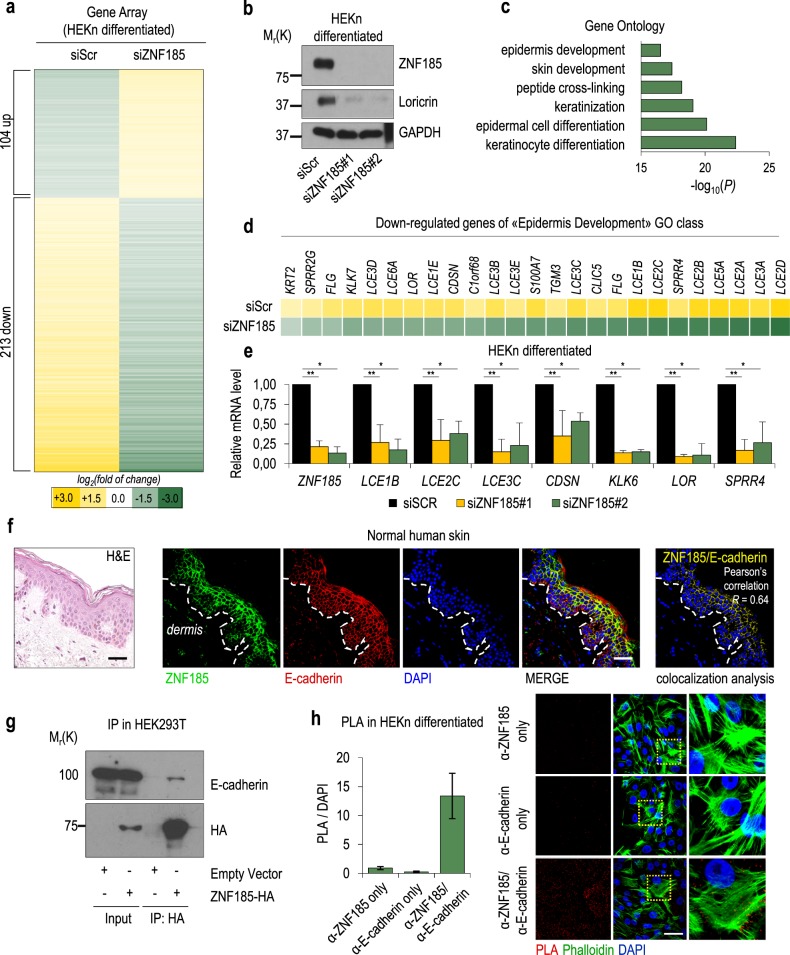
ZNF185 silencing causes delayed keratinocyte differentiation. **a** Heat map of gene expression after ZNF185 silencing by siRNA in differentiated HEKn by microarray analysis. *P* < 0.05; abs(*FC*) > 2. **b** Western blot analysis of ZNF185 and Loricrin levels in differentiated HEKn upon ZNF185 depletion with two different siRNA sequences. **c** GO terms for downregulated genes from (**a**). **d** Heat map of downregulated genes from the “Epidermis Development” group. **e** qPCR analysis of the mRNA level of several downregulated genes upon ZNF185 depletion in differentiated HEKn with two different siRNA sequences. *P < 0.05, *n* = 2, **P < 0.05, *n* = 3. **f** Immunofluorescence analysis of ZNF185 and E-cadherin levels in human epidermis. Scale bar: 100 µm. Colocalization of ZNF185 and E-cadherin was evaluated using Nikon EZ C.1 software. Pearson’s correlation *R* = 0.64. **g** Immunoprecipitation of ZNF185 with anti-HA antibody. Western blot for E-cadherin and HA. **h** PLA analysis of ZNF185 and E-cadherin interaction in differentiated HEKn. Staining with either anti-ZNF185 alone or anti-E-cadherin alone antibodies was performed as control. Scale bar: 50 µm. Histogram showing one representative experiment. PLA signal counts were normalized by DAPI signal counts. Ten different fields were analysed for each sample

To investigate the impact of ZNF185 depletion during keratinocyte differentiation, we performed a gene array in differentiated HEKn after RNAi-mediated knockdown of ZNF185. We identified 104 upregulated and 213 downregulated genes (cut-off abs(FC) > 2) (Fig. 4a, b, Supplementary Table S3). GO analysis of upregulated genes did not give any significant distribution (data not shown); meanwhile, downregulated genes were classified in different groups linked to epidermis development (Fig. 4c, Supplementary Table S5). Interestingly, many genes from the “epidermis development” group are situated in the epidermal differentiation complex (EDC) locus and were downregulated (Fig. 4d). Gene array data were validated by RT-qPCR (Fig. 4e). Loricrin downregulation was also shown at the protein level (Fig. 4b). Furthermore, the ZNF185 expression pattern in human tissues parallels the expression of genes related to the “keratinization” and “establishment of skin barrier” categories (Fig. S3f). These data demonstrated that ZNF185 knockdown delays keratinocyte differentiation.

Since cell adhesion and adherens junctions-mediated actin cytoskeleton dynamics plays an important role in keratinocytes during epithelia stratification and differentiation, we explored the possibility that ZNF185 could be part of these crucial biological processes. By studying publicly accessible mass spectrometry data, we found that ZNF185 could be a putative interactor of E-cadherin [33], a major component of the adherens junctions. Immunofluorescence analysis of the human epidermis revealed colocalization of ZNF185 and E-cadherin (Pearson’s correlation *R* = 0.64) (Fig. 4f). ZNF185/E-caderin interaction was also confirmed at the semi-endogenous level by co-immunoprecipitation in HEK293T cells overexpressing ZNF185 HA-tagged (Fig. 4g). Furthermore, proximity ligation assay (PLA) experiments revealed ZNF185 and E-cadherin interaction in differentiated keratinocytes (Fig. 4h). Altogether, these results indicate that ZNF185, interacting with E-cadherin, contributes to tissue formation and stability within stratified epidermis.

### ZNF185 is downregulated in SCC

**Fig. 5:**
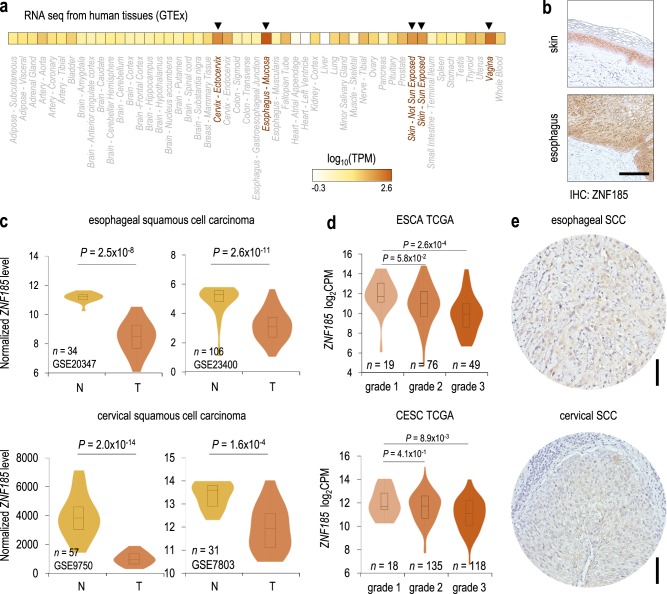
ZNF185 is downregulated in oesophageal and cervical SCC. **a** Heat map showing average *ZNF185* expression values (log_10_TPM) in different human tissues from the GTEx portal. **b** Immunohistochemical analysis of ZNF185 expression in normal skin and oesophagus. Scale bar: 100 µm. **c** Violin plots show *ZNF185* mRNA level in normal (N) or tumour SCC (T) samples determined by microarray analysis. Different datasets from the GEO database were analysed. **d** Violin plots showing *ZNF185* mRNA level in the samples of different grades from the TCGA ESCA and CESC panels determined by RNA-seq. **e** Immunohistochemical analysis of ZNF185 expression in oesophageal and cervical squamous cell carcinoma samples. Scale bar: 100 µm

Many studies have reported that adherens junctions and cell–cell adhesion in general are key factors for maintenance of epithelial homeostasis and play a role in regulating cell migration and tumour formation. Indeed, loss of E-cadherin, a key component of the adherens junctions, is associated with tumour development and epithelial–mesenchymal transition (EMT) connected with tumour cell invasion [34]. Since ZNF185 physically interacts with E-cadherin, we asked whether ZNF185 could also be modulated in squamous cell carcinomas. Firstly, we analysed *ZNF185* expression at mRNA level in normal tissues using GTEx portal. As shown in the heatmap in the Fig. 5a, besides skin, the tissues with the highest *ZNF185* expression are the oesophagus, vagina and cervix. We confirmed these findings by immunohistochemistry staining of normal oesophagus, which showed a similar pattern of *ZNF185* expression with respect to the normal skin (Fig. 5b). Thus, we analysed different datasets of oesophageal (GSE20347 and GSE23400) and cervical (GSE9750 and GSE7803) squamous cell carcinoma. In all cases we saw a significant decrease (*p*-value: 1.6 × 10^−4^–2.0 × 10^−14^) of *ZNF185* expression in tumour samples with respect to the normal tissues (Fig. 5c). Similarly, oesophageal squamous cell carcinoma (ESCA) and cervical squamous cell carcinoma (CESC) data sets from TCGA showed a similar trend, with a significant decrease (*p*-value: 4.1 × 10^−1^–2.6 × 10^−4^) of *ZNF185* expression in the high-grade tumours with respect to the low-grade tumours in both types of carcinomas (Fig. 5d). Analysis of oesophageal and cervical squamous cell carcinoma confirmed ZNF185 downregulation at protein level (Fig. 5e).

**Fig. 6:**
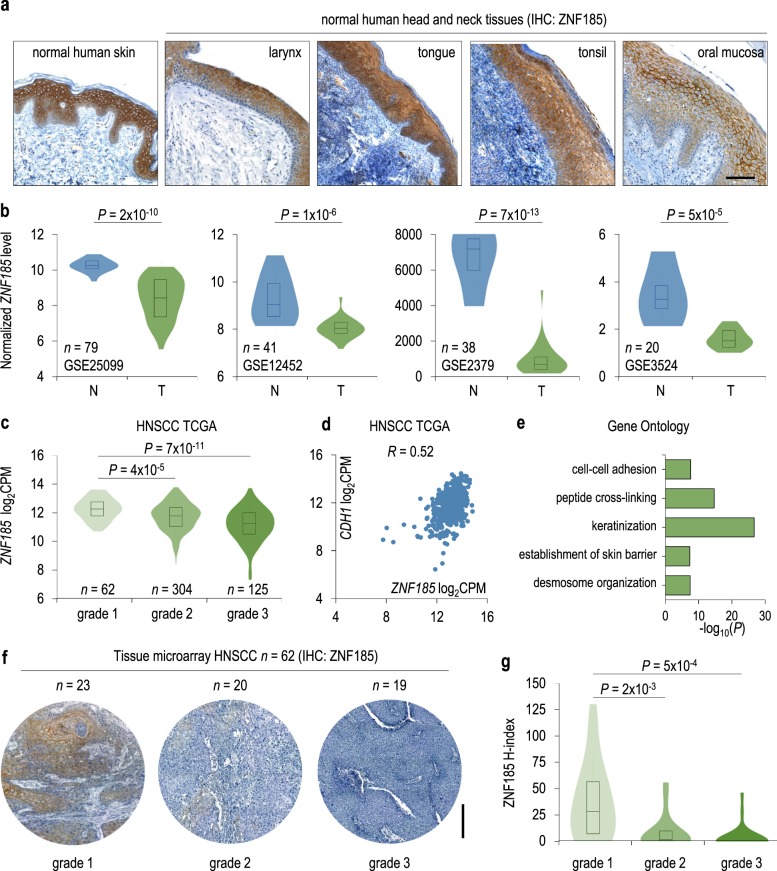
ZNF185 is downregulated in HNSCC. **a** Immunohistochemical analysis of ZNF185 expression in normal skin and head and neck tissues. Scale bar: 100 µm. **b** Violin plots show *ZNF185* mRNA level in normal (N) or tumour HNSCC (T) samples determined by microarray analysis. Different datasets from the GEO database were analysed. **c** Violin plot showing *ZNF185* mRNA level in the samples of different grades from the TCGA HNSCC panel determined by RNA-seq (*n* = 520). **d** Dot plot showing the correlation between the mRNA levels of *ZNF185* and *CDH1* in the TCGA HNSCC panel (*n* = 520, *R* = 0.52). **e** GO terms for genes whose mRNA level is correlated with the *ZNF185* mRNA level in the TCGA HNSCC panel (*n* = 520, *R* > 0.5). **f** ZNF185 expression in tissue microarray immunohistochemical analysis of different grades HNSCC samples. Scale bar: 500 µm. **g** Violin plot showing the distribution of H-index for ZNF185 level in the samples from (**f**)

Given that ZNF185 is expressed both in the epidermis and in head and neck normal tissues (Fig. 6a), we also analysed the *ZNF185* mRNA level in different HNSCC sample datasets (GSE25099, GSE12452, GSE2379, and GSE3524) and found a significant (*p*-value: 5 × 10^−5^–7 × 10^−13^) decrease in *ZNF185* expression in tumour samples compared to normal tissue (Fig. 6b). Analysis of the TCGA dataset of HNSCC revealed a sequential decrease from HNSCC grade 1 to grade 3 (*n* = 520, *p*-value: 4 × 10^−5^–7 × 10^−11^) (Fig. 6c). Since ZNF185 is p63 transcriptional target, we decided to analyse the ZNF185–p63 link in the HNSCC samples. However, we did not find a strong correlation with *TP63* expression (Figure S4a–b) in HNSCC datasets, despite the data shown in normal keratinocytes, indicating that, although *TP63* is upregulated, additional genetic and epigenetic changes affect its activity in SCC cells. Interestingly, in line with previous reports demonstrating that the expression of *CDH1*, e-cadherin coding gene, is reduced in epithelial cancers (Figure S4c–d) [34], we detected a positive correlation between the expression of *ZNF185* and *CDH1* (Pearson’s correlation *R:* 0.52 ÷ 0.80, Fig. 6d, Figure S4e). This link is further supported by the GO analysis showing that *ZNF185* expression in HNSCC is correlated with the expression of other genes important for keratinization and desmosomal organization (Fig. 6e, Supplementary Table S5). To further validate the correlation of the ZNF185 expression pattern at the protein level in grade 1–grade 3 tumour samples, we performed immunohistochemistry (IHC) staining of ZNF185 in a tissue tumour microarray containing 62 samples of human HNSCC. We confirmed decreased membrane staining of ZNF185 (*p*-value: 2 × 10^−3^ and 5 × 10^−4^) in poorly differentiated tumour cells compared to well-differentiated tumour cells and normal cells of the squamous epithelium (Fig. 6f, g).

Altogether, these data demonstrate that *ZNF185* expression is dramatically downregulated in the high-grade poorly differentiated SCC tumours, confirming that deregulation of cell adhesion programmes plays a crucial role in epithelial tumour formation. ZNF185 could be included as a prognostic marker to assess the differentiation state in oesophageal, cervical and head and neck SCC tumours.

## Discussion

*ZNF185* gene codifies for an actin-cytoskeleton-associated Lin-l 1, Isl-1 and Mec-3 (LIM) domain-containing Zn-finger protein and was originally isolated on chromosome Xq28 [35], however, its function is poorly known [36]. At the mRNA level, *ZNF185* is detected in several normal human tissues, including the prostate [37]. The actin-targeting domain is located at the N-terminus, and it is necessary to mediate actin-cytoskeleton targeting of ZNF185, while the LIM domain, which is localized in the C-terminus, is dispensable for actin binding [38]. The LIM domain is a protein–protein interaction domain present in a wide range of proteins whose functions are related to cytoskeleton dynamics, development and cell lineage specification [39–41], but it is also involved in pathologies such as cancer [38]. Epigenetic silencing of *ZNF185* has been associated with high-grade and metastatic prostate tumours [36], lung tumours, and head and neck squamous cell carcinomas [42, 43]. Downregulation of ZNF185 expression seems to be a frequent event in several tumour types, suggesting that *ZNF185* acts as a tumour-suppressor gene [44].

ZNF185 appears to contribute to the p63-related differentiation sub-programmes in normal keratinocytes. Indeed, using human keratinocytes and normal human epidermis as a model system, we identified the functional link between p63 and the new target gene, *ZNF185*. By bioinformatic tools, RNA sequencing, and ChIP sequencing data sets, we analysed the genomic region and studied the transcriptional regulation of ZNF185 expression. We demonstrated the presence of a new promoter P0 located 15 kb up-stream of its annotated promoter, and a skin-specific p63 enhancer located 20 kb up-stream of the P0 promoter. ZNF185 function in epithelia has been poorly studied. As with some other Zn-finger proteins from the LIM-domain family [45], ZNF185 is localized in both the cytoplasm and the cellular periphery. We showed that ZNF185 increases during differentiation and that its depletion delays differentiation and downregulation of subsequent differentiation markers (EDC genes), confirming that correct stratification in keratinocytes relies on cell–cell adhesion and actin-cytoskeleton dynamics [2, 46]. The limited range of p63 and ZNF185 co-expression in cells and normal epidermis indicates that ZNF185 belongs to the p63 target genes sub-set required for the proliferation/differentiation switch (ie. Notch1, JAG1, Hes1) [47, 48] and to maintain epithelial adhesion and integrity of the differentiated upper layers (ie. PERP, EVPL, BPAG1) [9, 49, 50]. Furthermore, ZNF185 physically interacts with E-cadherin, a component of the adherens junctions and one of the critical cell–cell adhesive complexes, which include tight junctions and desmosomes, of the pluristratified epithelia.

**Fig. 7:**
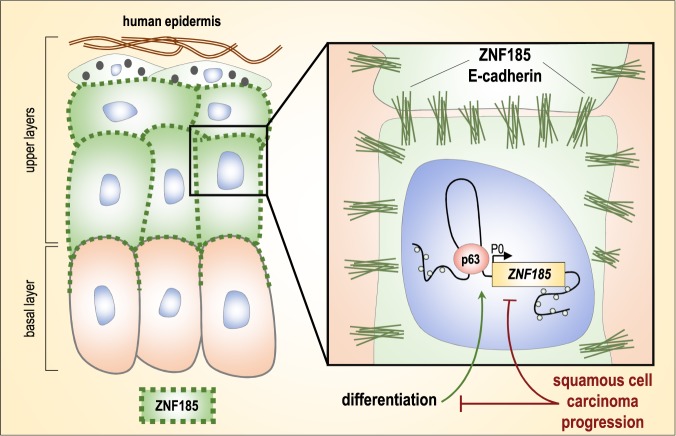
A proposed model of the p63-ZNF185 functional link in epidermal development and cancer. p63 maintains the proliferation compartment of the epidermis and influences the early steps of stratified epithelial formation by direct transcriptional activation of selected cell-adhesion-related genes, including *ZNF185*. Expression of ZNF185 is required in vitro to express late differentiation marker genes. In SCC, ZNF185 expression is strongly reduced, and it is undetectable in poorly differentiated cancer cells. This represents an example of p63 activity deregulation in cancer specific cells

p63, specifically the ΔNp63 isoform, is frequently overexpressed in various carcinomas, including head and neck squamous cell carcinomas (HNSCC) [51–53]. Unexpectedly, we observed that the transcription of *ZNF185* is strongly reduced in HNSCC tumours in parallel with the E-cadherin decrease. A weak correlation between p63 and ZNF185 expression indicates that additional genetic and epigenetic regulators change the p63 transcriptional signature in SCC cells as compared to normal epithelial cells. ZNF185 expression is undetectable at the protein level in poorly differentiated tumour cells of HNSCC tissues compared to well-differentiated tumour cells and normal tissues. Similar results occur in oesophageal and cervical SCC. The importance of altered cell–cell adhesion is commonly observed during human tumour initiation and progression [54]. Several genetic experiments performed in mice have demonstrated that murine knockout of E-cadherin, p120-catenin and other adherens junction components can contribute to cancer development [22–24, 55–57]. Our findings indicate that ZNF185 is part of the deregulated programme that affects SCC cancer cell behaviour (Fig. 7). Our data indicate that while a decrease in E-cadherin is generally a useful prognostic biomarker in different epithelial tumour types, loss of ZNF185 is strongly associated with late tumour stages and poorly differentiated head and neck, possibly oesophageal and cervical, SCC tumours.

Our studies expand the knowledge about the role of p63 in epithelial biology and identify a new factor, ZNF185, that could improve cancer diagnosis, prognosis and therapy. Further studies on the diagnostic and prognostic value of ZNF185 in cancer are warranted.

## Materials and methods

### Cell culture, transfection and treatments

Neonatal human epidermal keratinocytes (HEKn, Life Technologies, Carlsbad, CA, USA) and immortalized human epidermal keratinocytes Ker-CT (ATCC, Manassas, VA, USA) were cultured in EpiLife medium with human keratinocyte growth supplements (HKGS, Life Technologies). HEK293T and H1299 cells were grown in DMEM medium (Lonza, Basel, Switzerland) with the addition of 10% FBS, 100 U penicillin, and 100 μg/mL streptomycin (Gibco, Life Technologies). Then, 2.5 × 10^5^ cells were transfected with 80 pmol of specific siRNAs (Supplementary Table 1) by Lipofectamine RNAiMAX Transfection Reagent (Invitrogen, Carlsbad, CA, USA) and collected 48 h post transfection. HEKn or Ker-CT cells were differentiated in vitro by adding 1.2 mM CaCl_2_ to culture medium. Cells were collected at the indicated times or at 3 days of differentiation if not indicated otherwise. Further details for the generation of 3D organotypic skin model equivalents are described in Supplementary Material and Methods.

### RNA sequencing

Total RNA was extracted using a mirVana miRNA isolation kit (Thermo Fisher). rRNA was removed from each RNA extraction before proceeding with RNA seq library construction. Sequencing was performed on an SOLiD sequencer 5500XL (Applied Biosystems) with 75-base-pair single-end reads. See supplemental information for discussion of RNA seq analysis methods. Further details for RNA extraction and RT-qPCR analysis, bioinformatic analysis, analysis of the *ZNF185* genomic locus, gene expression microarray, chromatin conformation capture (3C) assay, chromatin immune-precipitation assay and luciferase activity assay are described in Supplementary Materials and Methods.

### ZNF185 cloning

Total RNA (1 µg) extracted from HEKn at 3 days of differentiation was retrotranscribed using oligo(dT) primers. ZNF185 cDNA was amplified by PCR using specific primers (Supplementary Table 1). ZNF185 cDNA was subcloned into pcDNA3.1-HA vector (Invitrogen) and completely sequenced.

### Western blot

HEKn and Ker-CT cells were lysed in SDS lysis buffer (100 mM Tris рН 8.8, 1% SDS, 5 mM EDTA, 20 mM DTT, and 2 mM AEBSF). Total protein extracts were resolved in SDS polyacrylamide gel and blotted onto a Hybond PVDF membrane (GE Healthcare, Chicago, IL, USA). The following antibodies were used: anti-Loricrin (1:1000, Covance, Princeton, NJ, USA), anti-ZNF185 (1:300, Sigma), anti-Keratin-10 (1:1000, Covance), anti-E-cadherin (1:1000, Cell Signaling, Danvers, MA, USA), anti-GAPDH (1:15000, Sigma), anti-p63alpha (1:500, D2K8X, Cell Signaling), and anti-HA (1:1000, BioLegend, San Diego, CA, USA). Uncropped images of western blots from this study are shown in Figure S6. Further details for western blot, immunohistochemical staining and TMA are described in Supplementary Materials and Methods.

### Immunoprecipitation

HEK293T cells (1 × 10^7^) were transfected with 20 µg of either pcDNA3.1-HA-ZNF185 or pcDNA3.1-HA (EV, as negative control) vectors using Lipofectamine 2000 according to the manufacturer’s instructions (Invitrogen). Twenty-four hours after transfection, cells were collected and lysed in Triton buffer (50 mM Tris pH7.5, 150 mM NaCl, mM EDTA, 0.5% (v/v) Triton X-100, 1 mM DTT, 0.1 mM PMSF, 1 mM sodium orthovanadate, and 1x complete protease inhibitors (Roche, Basel, Switzerland)). Total proteins (5 mg) were immunoprecipitated using 5 µg of anti-HA antibody (Covance). Total protein extracts (30 µg) were loaded as input fractions. Further details for Proximity ligation assay (PLA) are described in Supplementary Materials and Methods.

### Statistical analysis

The significance of differences between two experimental groups was calculated using the two-tailed Student’s t-test. Values with *P* < 0.05 were considered significant. For the RT-qPCR and luciferase assay, values reported are the average ± SD of three independent experiments. Violin plots were generated using BoxPlotR web-tool (http://boxplot.tyerslab.com/).

### Accession numbers

The gene expression microarray data have been deposited in the NCBI Gene Expression Omnibus with accession number GSE102613.

## Electronic supplementary material

Supplementary Figures

Supplementary Materials and Methods

Supplementary Table 1 - primer sequences

Supplementary Table 2 - RNA seq

Supplementary Table 3 - Gene Array

Supplementary Table 4 - data sets

Supplementary Table 5 - GO analyses
