# Supplementary material for: ZNF185 is a p63 target gene critical for epidermal differentiation and squamous cell carcinoma development
Source: Oncogene. 2018 Oct 18;38(10):1625–38. doi: 10.1038/s41388-018-0509-4 (PMC6755960; doi:10.1038/s41388-018-0509-4)
Supplement: Supplementary file 1 — Supplementary Figures [file 41388_2018_509_MOESM1_ESM.pdf]

SUPPLEMENTARY FIGURE S1 – *Smirnov et al.*

a

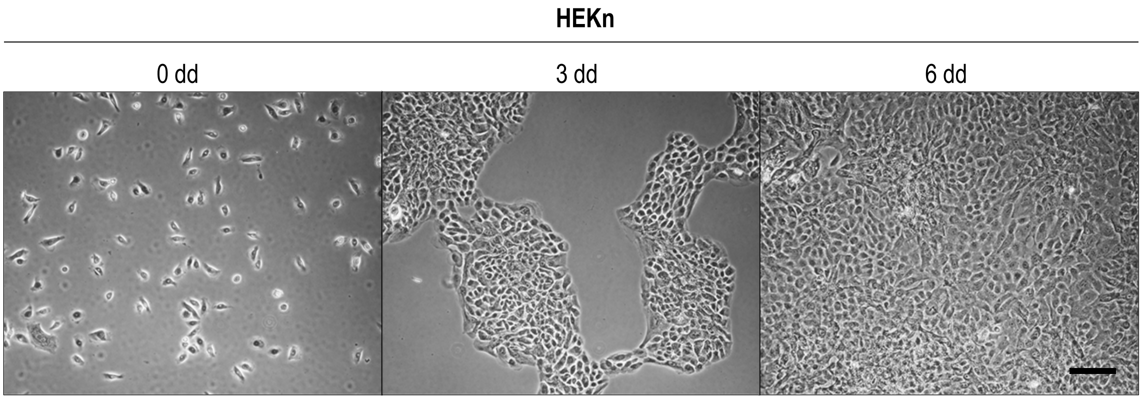

b

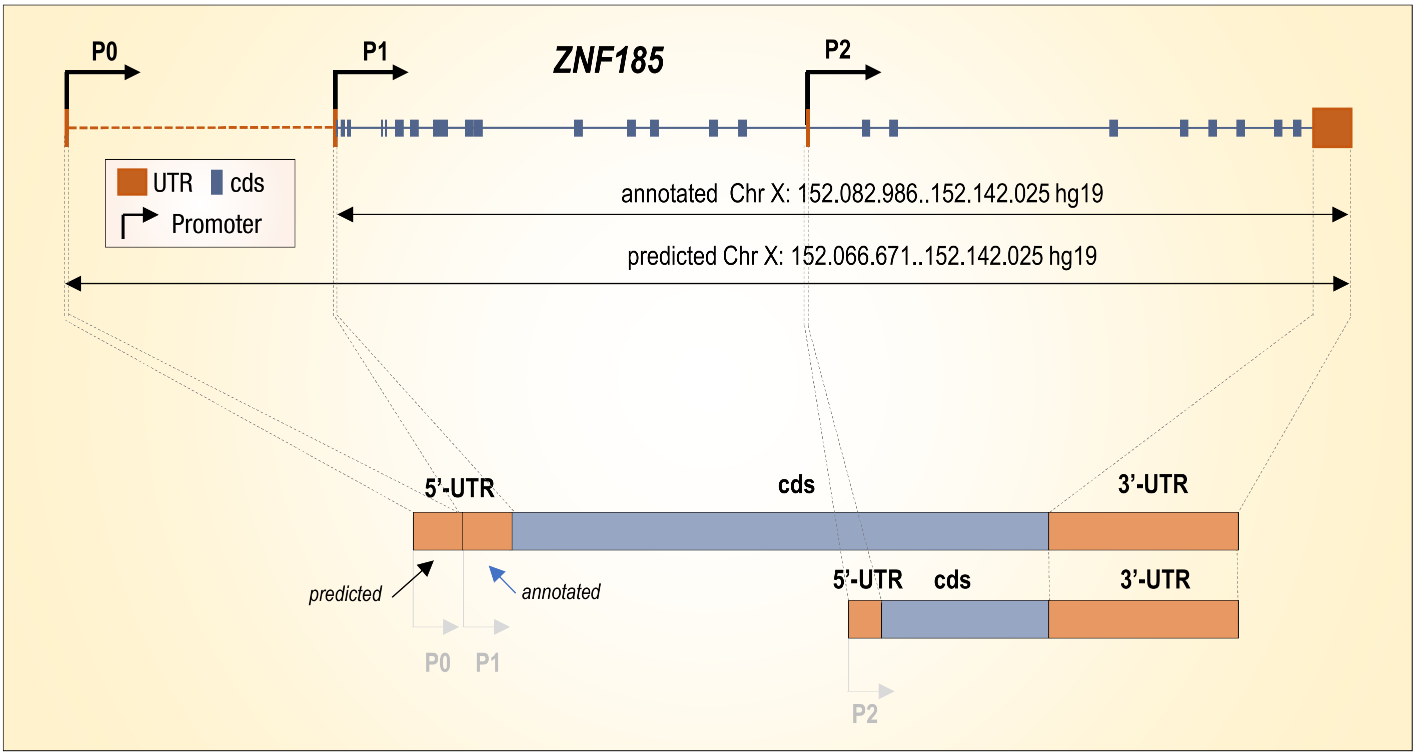

c

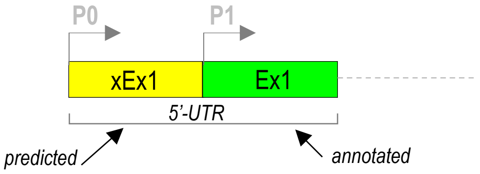

d

```
GTTTTCCATCACGGCAGAGCCTTCTGGAGCTCGCCCCGTGCTCGACAAGGG
GAGCCCCAGACTTGATTTCTCCAGCAGCCCCGCGGGGCGGGGCCGAGAAGG
GGCGGGCGGGCGGAGGACGCTGGCGGGAGAATGTGCGGAATGTGCCAGGCG
CCTCTACCTACCCCAGGAGGGGCAGAGGAAGGGGCCGGCCGAGGGGAGGGG
CGAGGGGCGAGGCTCCGGGCGGCCGGAGAAAACCTGTTTGTTCTCCTGCGG
GCGGAGCCCAGTCCGCGCCTCCTCGCCTCCCTGCGCGCTCCCCGCTGTGCA
CCGCGCCCCGCGCCCCGCGCCTGCCTCCCCGGCCCGGCCCGGCCCGGCCGG
GCGCCGTCCACCCGAGGGAGCCCGCGCCCTGCGACTGCCCGACCCTCCACC
CGCCCCACGAGCCGGCACCGGTGCCAGAAGCCCTGCTGAATCAACTGAGAA
GAGAGCTGCGGAGCAATCACCATGAGTATCTCAGCTCTGGAGGCCACACCA
AAGGAAGCCTCGACC
```

SUPPLEMENTARY FIGURE S2 – *Smirnov et al.*

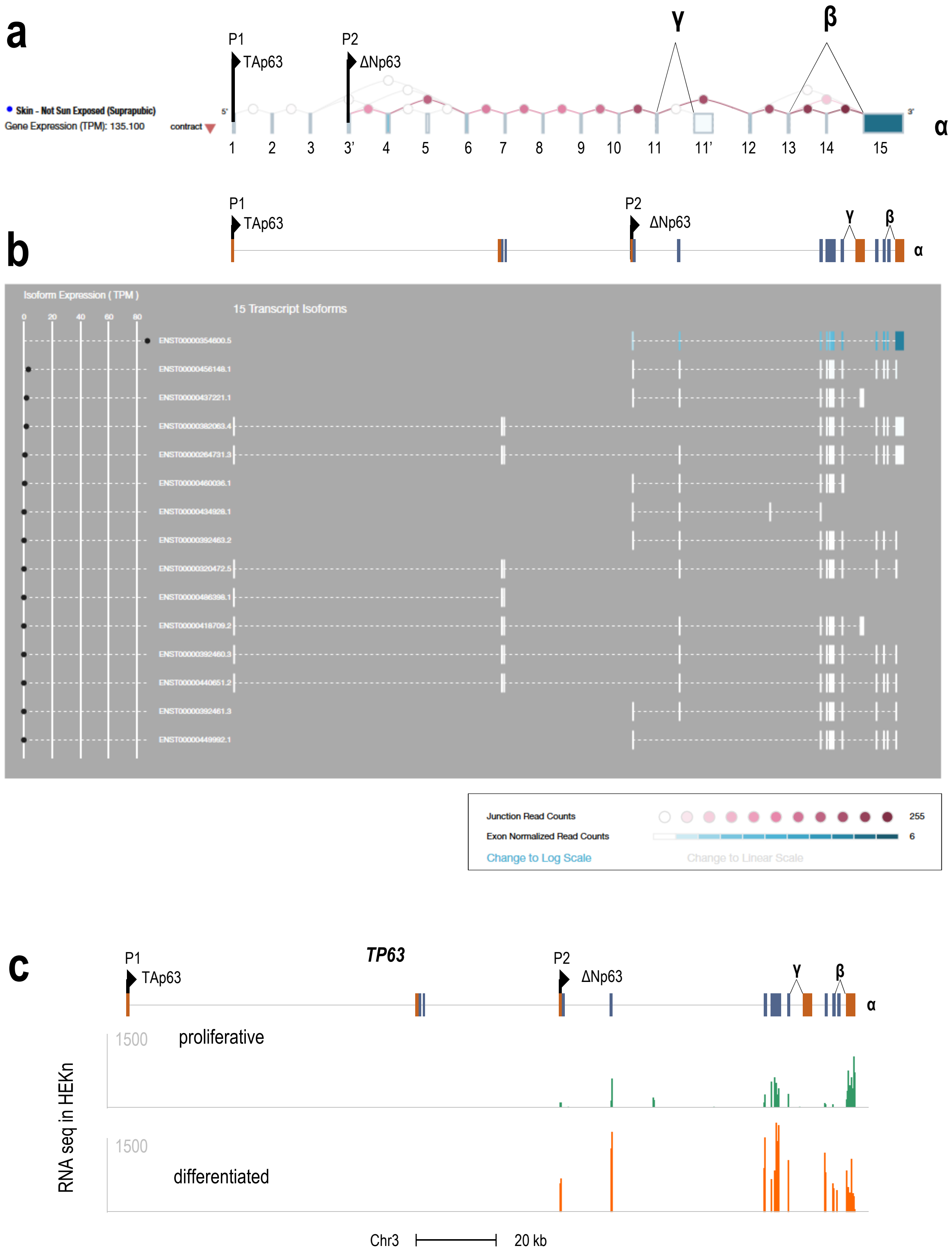

SUPPLEMENTARY FIGURE S3 – *Smirnov et al.*

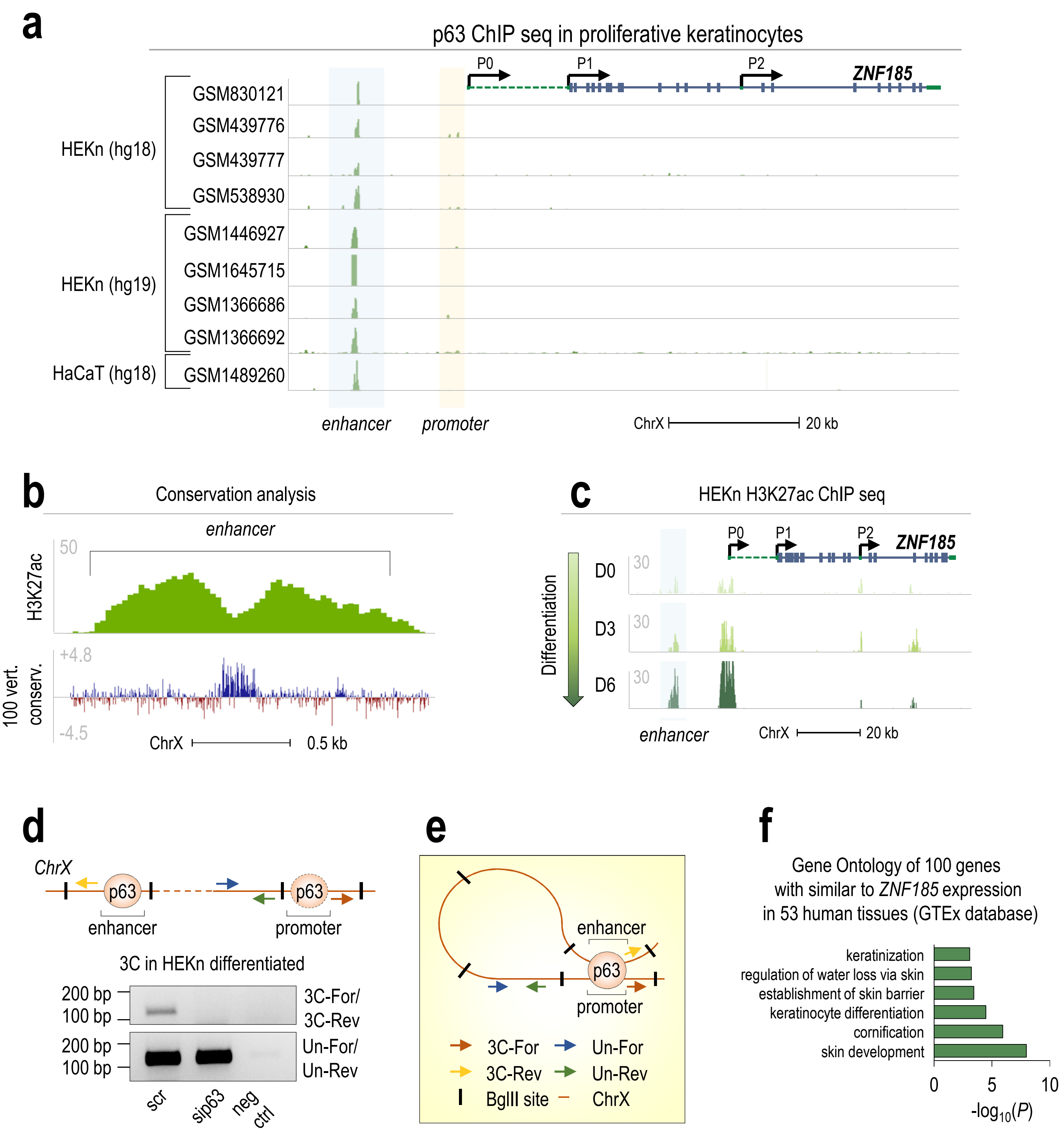

SUPPLEMENTARY FIGURE S4 – *Smirnov et al.*

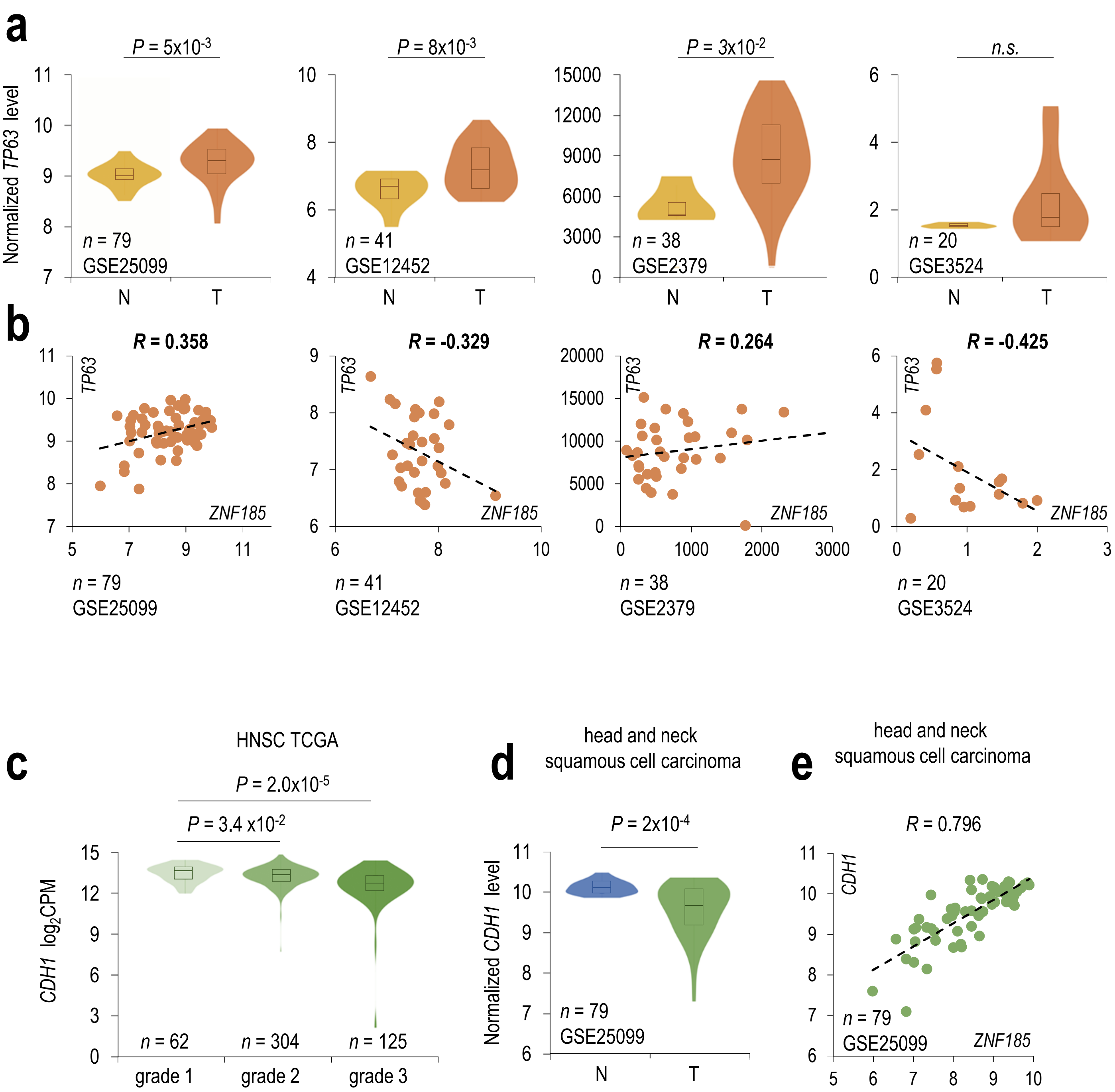

SUPPLEMENTARY FIGURE S5– *Smirnov et al.*

a

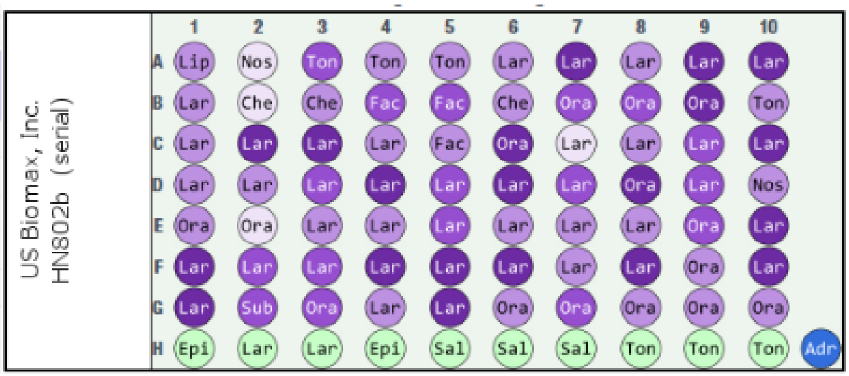

HNSCC TMA *n* = 62
IHC: ZNF185

b

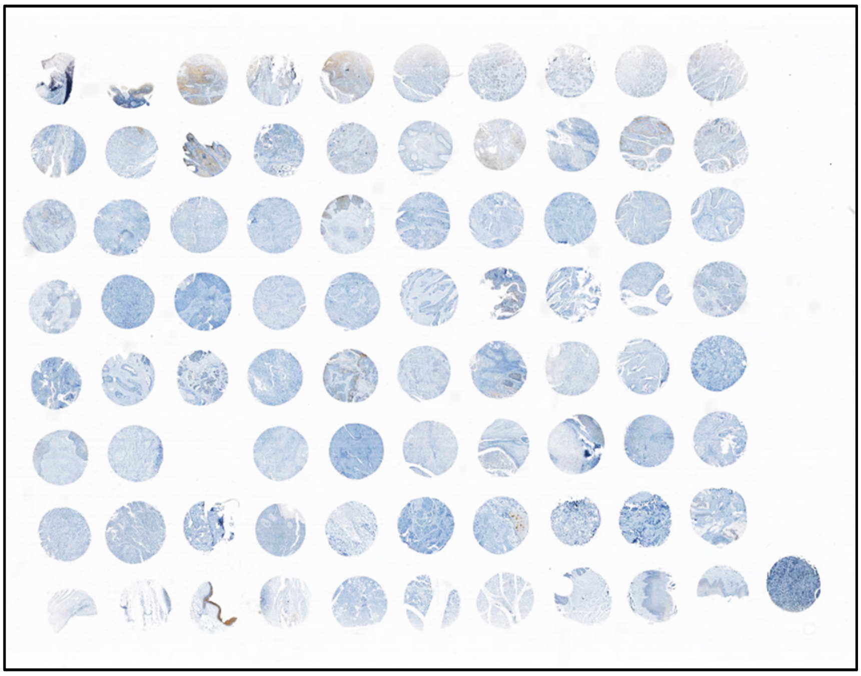

SUPPLEMENTARY FIGURE S6 – *Smirnov et al.*

WB supporting Fig. 2b

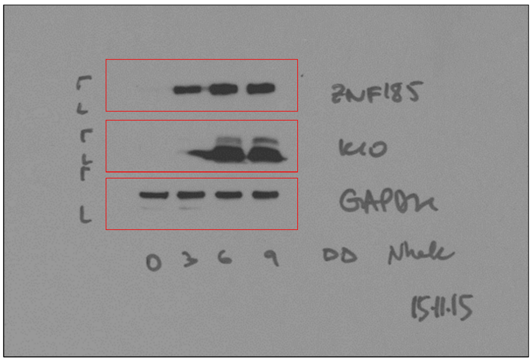

WB supporting Fig.3h

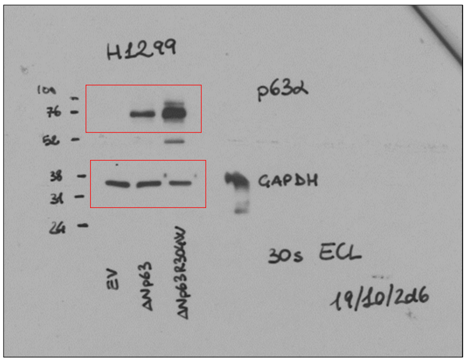

WB supporting Fig. 3i

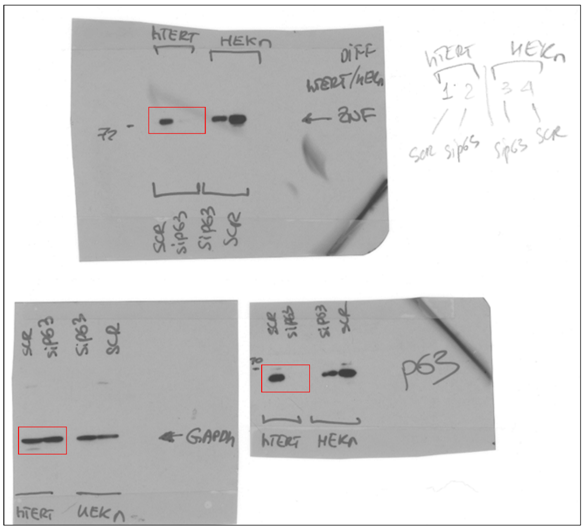

WB supporting Fig. 2c

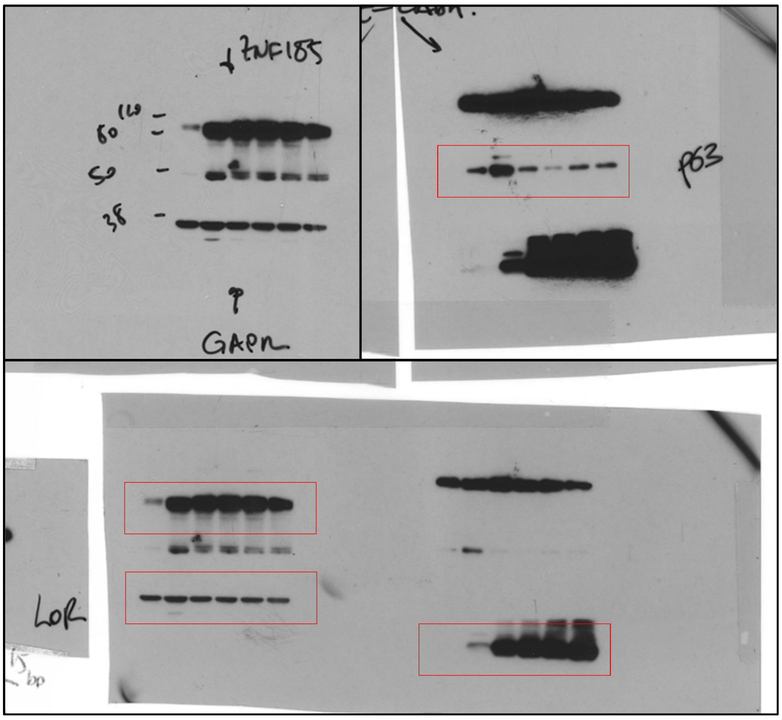

WB supporting Fig. 4b

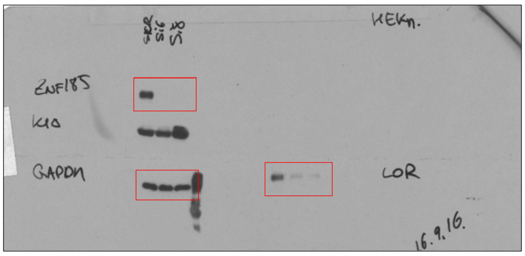

WB supporting Fig. 4g

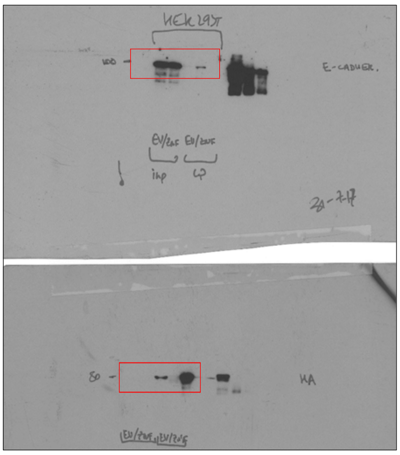

SUPPLEMENTARY FIGURE S7 – *Smirnov et al.*

IGB screenshot supporting Fig. 1b; 2a

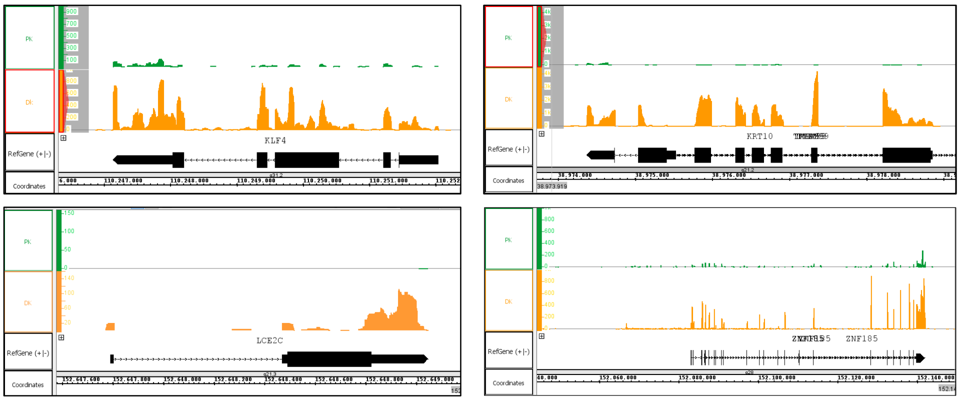

IGB screenshot supporting Fig. 3a

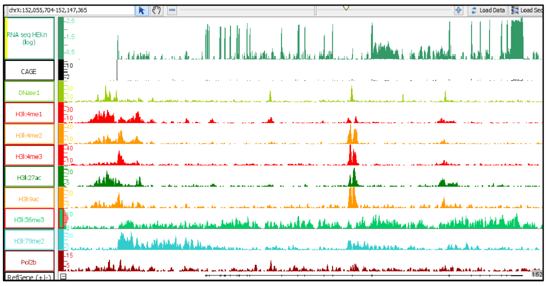

IGB screenshot supporting Fig. 3c

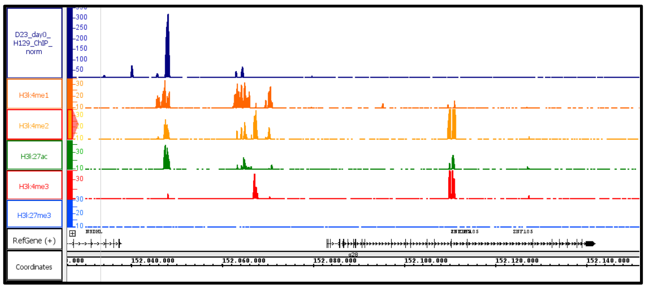

IGB screenshot supporting Fig. 3d

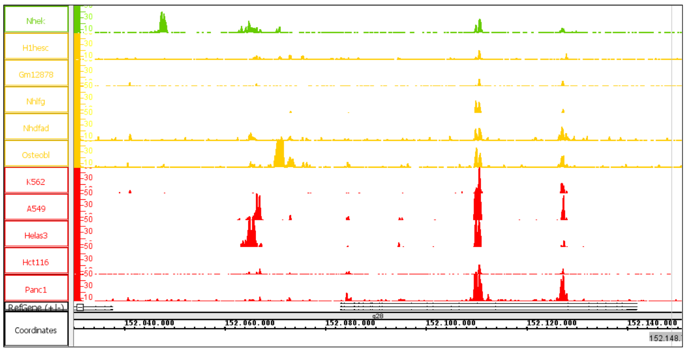

IGB screenshot supporting Fig. 3e

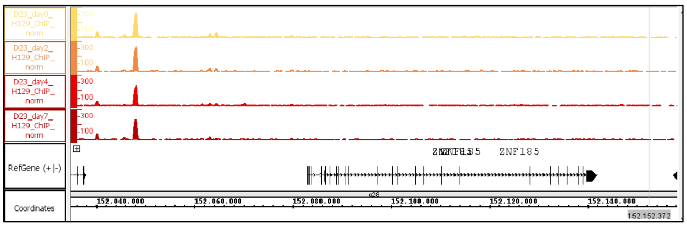

Supplementary Figure legends

Supplementary Figure S1
(a) Phase contrast microphotographs showing morphological changes of HEKn during differentiation**.** Scale bar: 200 μm. **(b)** A schematic representation of ZNF185 gene structure, its mRNA, and TSS location. **(c-d)** Sequencing result of amplification of predicted xEx1 within 5'-UTR of mRNA of *ZNF185* obtained from differentiated keratinocytes. Yellow indicates the newly identified exon-1 (xEx1) sequence, and green is the annotated exon-1 (Ex1).

Supplementary Figure S2
(a-b) Analysis of *TP63* expression in the human skin by GTEx portal. **(c)** *TP63* expression by RNA seq in proliferating and differentiated keratinocytes.

Supplementary Figure S3
(a) Genomic locus of the *ZNF185* showing the enhancer region with p63 ChIP-seq signal tracks from different studies. **(b)** Enhancer locus of *ZNF185* with H3K27ac ChIP-seq signal, including analysis of conservation in 100 vertebrates. **(c)** Enhancer locus of *ZNF185* with H3K27ac ChIP-seq signal enrichment at 0, 3, and 6 days of keratinocyte differentiation. **(d)** Fragments resulting from **a**mplification by PCR of the chromatin conformation capture (3C) assay performed in differentiated HEKn upon p63 depletion. BglII was used for chromatin digestion. To amplify the hypothetical loop region, 3C-For and 3C-Rev primers were used. An undigested region was used as a loading control after amplification using Un-For and Un-Rev primers. **(e)** Schematic representation of DNA loop between enhancer and promoter loci of the *ZNF185* gene. **(f)** GO terms for 100 genes with the same *ZNF185* expression pattern in 53 human tissues from the GTEx database

Supplementary Figure S4
(a) Violin plots show the *TP63* mRNA level in normal (N) or tumour HNSCC (T) samples determined by microarray analysis. Different datasets from the GEO database were analysed. **(b)** Dot-plot showing the correlation between the mRNA levels of ZNF185 and TP63 in the datasets from (a). (c-d) Violin plot show the *CDH1* mRNA level in normal (N) or tumour HNSCC (T) samples from TCGA HNSCC panel or from GSE25099 dataset. **(e)** Dot-plot showing the correlation between the mRNA levels of *ZNF185* and *CDH1* in the GSE25099 dataset.

Supplementary Figure S5
(a) Schematic representation of the position and description of samples of HNSCC tissue microarray. **(b)** Scanned slide with immunohistochemical staining of ZNF185 on a HNSCC tissue microarray**.**

Supplementary Figure S6
Uncropped images of western blots from the Fig 2b, Fig 2c, Fig 3h, Fig 3i, Fig 4b, Fig 4g. Red boxes highlight the cropped areas used in the main figures.

Supplementary Figure S7
Uncropped original screenshots of sequencing data visualized in Integrated Genome Browser (IGB) used in the Fig 1b, Fig. 2a, Fig 3a, Fig 3c, Fig 3d, Fig 3e.
