# Supplementary material for: ZNF185 is a p63 target gene critical for epidermal differentiation and squamous cell carcinoma development
Source: Oncogene. 2018 Oct 18;38(10):1625–38. doi: 10.1038/s41388-018-0509-4 (PMC6755960; doi:10.1038/s41388-018-0509-4)
Supplement: Supplementary file 2 — Supplementary Materials and Methods [file 41388_2018_509_MOESM2_ESM.docx]

*Smirnov et al.*

**Supplementary Materials and Methods**

**Western blot**

Membranes were blocked with 5% non-fat dry milk (Bio-Rad, Hercules, California, USA) in 0.1% Tween-20 solution in PBS at room temperature for 1 h. They were incubated with primary antibodies at +4 °C overnight and with the goat anti-rabbit and goat anti-mouse appropriate horseradish peroxidase-conjugated secondary antibodies (Bio-Rad) at room temperature for 1 h. Detection was performed with the ECL chemiluminescence kit (Perkin Elmer, Waltham, Massachusetts, USA).

**RNA extraction and RT-qPCR analysis**

Total RNA was isolated using the RNeasy Mini Kit (Qiagen, Venlo, The Netherlands) according to the manufacturer’s protocol. Total RNA (1 µg) was used for cDNA synthesis by GoScript Reverse Transcription System kit (Promega, Madison, WI, USA). RT-qPCRs were performed using the GoTaq Real-Time PCR System (Promega) in Applied Biosystems 7500 Real-Time PCR System (Applied Biosystems, LifeTechnologies) using appropriate qPCR primers (Supplementary Table 1). *TBP* was used as a housekeeping gene for normalization. The expression of each gene was defined from the threshold cycle (C_t_), and relative expression levels were calculated using the 2^−ΔΔCt^ method. All reactions were run in triplicate.

**Analysis of the *ZNF185* genomic locus**

To analyse the *ZNF185* genomic locus in human keratinocytes, different publicly accessible high-throughput sequencing data were used. Precisely, CAGE-seq (Cap Analysis Gene Expression, it measures RNA expression and maps TSS in promoters), DNase-seq (Sequencing of DNase I hypersensitive sites, to identifying cis-regulatory elements across the genome), ChIP-seq for Pol2, H3K4me1, H3K4me2, H3K4me3, H3K27ac, H3K9ac, H3K36me3, and H3K79me2 (histones modification enhancers and open chromatin marks) were downloaded from the ENCODE portal and visualized in the Integrated Genome Browser [55]. The ChIP-seq data from the NCBI GEO database were analysed to assess p63 binding to the *ZNF185* enhancer locus (**Fig. 3c, 3e**: GSM1446927, GSM1446928, GSM1446929, and GSM1446930; **Figure S3a**: GSM830121, GSM439776, GSM439777, GSM538930, GSM1645715, GSM1366686, GSM1366692, and GSM1489260). H3K27ac ChIP-seq data were used for time-course analysis of chromatin state (**Figure S3c**: GSM2247244, GSM2247245, and GSM2247246). All the experiments taken into account were carried out in the human normal neonatal keratinocytes grown in Keratinocyte Growth Medium with supplements. These conditions are compatible with the one used in our laboratory. More detailed description of experimental procedures can be found on the GEO NCBI web site under accession numbers indicated above. The conservation analysis of *ZNF185* enhancer locus was performed within the UCSC genome browser. To confirm the presence of a new exon *xEx1*, a specific 550 bp PCR product obtained after amplification was completely sequenced. To identify putative p63 binding sites, we used the “p53/p63 scan” software [28]. Uncropped screenshots of Integrated Genome Browser from this study are shown in the **Fig. S7**.

**Chromatin immunoprecipitation assay**

Ker-CT cells (1x10^6^) that were differentiated *in vitro* for 3 days were used for ChIP assay. Cells were fixed by 1% formaldehyde incubation, lysed and subjected to sonication for DNA shearing. Chromatin immunoprecipitation was performed with a specific anti-p63α antibody (Cell Signaling, D2K8X) or unspecific immunoglobulin G antibody (IgG, Invitrogen) using the MAGnify ChIP Kit (Invitrogen). Specific primers were used to amplify the identified putative p63 response element (**Supplementary Table 1**).

**Luciferase activity assay**

The enhancer region of *ZNF185* containing the putative p63 responsive element was amplified from human genomic DNA using specific primers (**Supplementary Table 1**), subcloned into the pGL3-promoter reporter vector (Promega) and completely sequenced. For luciferase assays,1x10^5^ H1299 cells were transfected with 100 ng of pGL3-promoter-*ZNF185* reporter vector, 2 ng of pRL-CMV-Renilla luciferase vector (Promega) and 300 ng of either pcDNA3.1-HA-ΔNp63α, pcDNA3.1-HA-ΔNp63α-R304W, or empty pcDNA3.1-HA vector (as a control) using Effectene according to the manufacturer’s protocol (Qiagen). Luciferase activity was measured 24 h after transfection using the Dual Luciferase Reporter Assay System (Promega) and light emission was measured over 10 sec using a Lumat LB9507 luminometer (EG&GBerthold, Bad Wildbad, Germany). The transfection efficiency was normalized to Renilla luciferase activity. Overexpression of the p63 protein was confirmed by Western blot.

**Proximity ligation assay (PLA)**

HEKn cells were seeded on 5 mm coverslips and differentiated *in vitro* for 3 days. Cells were fixed, permeabilized and incubated with primary antibodies as described. For labelling and PLA signal amplification, the Duolink In Situ Red Starter Kit Mouse/Rabbit kit (Sigma) was used according to the manufacturer’s protocol.

**RNA sequencing**

Paired-end sequencing of 2 × 75 bp was performed by Genomnia s.r.l. (Milan, Italy) using a SOLiD Sequencer 5500XL (Applied Biosystems). The sequencing was performed to a target of 100 million reads/sample (actual mapped reads ranged from 69-87M reads/sample). Sequencing reads in SOLID “xsq” format were mapped to the hg19 genome built and analysed with the Lifetech Lifescope 2.5.1 Whole Transcriptomic analysis pipeline with the Integromics Seqsolve software and proprietary Genomnia procedures. Reads per kilobase per million (RPKM) was assigned to each gene, then analysed between the corresponding proliferating or differentiated strata. All the results were manually revised, sorted and annotated.

Enriched pathways were identified in the up- and down- regulated genes using the Reactome public pathway database and its associated software tools (http://www.reactome.org/ReactomeGWT/entrypoint.html). Statistical analysis was performed with the Bioconductor edgeR program on the R statistical software (R version 2.15.2). The difference between the rank-ordered plot of enrichment scores for all genes is displayed in Fig. 1c. We did not performed a replicate of this experiment, however the enriched expressed genes in the two conditions were validated by comparing gene expression profiles taken by previous published results obtained from our and other laboratories (Fig.1b; Smirnov et al, 2016; Viticchiè et al, 2015; Sun et al, 2015; Kouwenhoven et al, 2015).

**Immunohistochemical staining and TMA**

FFPE sections (5 µm) were dewaxed and rehydrated. Immunohistochemical staining was performed using the BenchMark ULTRA slide staining system (Roche). For antigen retrieval, samples were incubated at 95 °C for 76 min in Cell Conditioning solution CC1 (Roche). Samples were incubated with anti-ZNF185 antibody (1:100, Sigma, Saint Louis, MO, USA) for 40 min. Sections were counterstained with Mayer’s haematoxylin, dehydrated and mounted using Bio Mount HM (BioOptica, Milan, Italy). The HNSCC tissue microarray section (US Biomax, Rockville, MD, USA) was stained for ZNF185 as described before. Samples were scored in a blinded manner by a pathologist using a semi-quantitative method. Cases were analysed for staining intensity, which was scored as 0 (not detected), 1+ (weak), 2+ (intermediate), and 3+ (strong). For each case, the H-index was calculated by multiplying the percentage of positive cells (0%-100%) by the intensity (0-3). All the tissues are obtained from adult donors. The samples from this study were utilized with the approval of the institutional review board of University Hospital “Policlinico Tor Vergata” (Rome, Italy) and prior patient consent.

**Immunofluorescence**

FFPE sections (5 µm) were dewaxed and rehydrated. Samples were boiled in 10 mM sodium citrate buffer pH 6.0 for 15 min for antigen retrieval. Sections were incubated for 1 h in 10% goat serum in PBS at room temperature and overnight at 4 ºC with primary antibodies. The following antibodies were used: anti-ZNF185 (1:50, Sigma), anti-Keratin 14 (1:1000, Covance), anti-Keratin 10 (1:1000, Covance), anti-E-cadherin (1:200, BD, Franklin Lakes, New Jersey, USA), and anti-pan-p63 (1:200, ab735, Abcam, Cambridge, UK). Sections were incubated for 1 h at room temperature with secondary anti-mouse and anti-rabbit 488- or 568-AlexaFluor conjugated antibodies (1:1000, Invitrogen) together with 1 μg/mL DAPI (Sigma) for nuclear DNA staining. Sections were analysed with a confocal laser microscope (NIKON Eclipse Ti) using EZ C.1 software (Nikon, Tokyo, Japan). HEKn cells were seeded on 5-mm coverslips, fixed for 10 min in 10% formalin buffered solution, washed with PBS and permeabilized in 0.2% Triton X-100 solution in PBS for 10 min. Blocking and incubation with antibodies were performed as described. For cytoskeleton staining, 488- or 568-AlexaFluor conjugated phalloidin was used (1:1000, Thermo Fisher, Waltham, Massachusetts, USA). For the 3D rendered confocal imaging of HEKn (**Fig 2d**), z-stacks were acquired every 0.1 µm for a total of 10 µm, and 3D rendering was performed with NIS elements software using the Alpha-blending algorithm.

**Bioinformatic analysis**

Gene ontology (GO) terms analysis was performed in gene sets using biological process annotation by Gene Ontology Consortium tools. Detailed GO analyses are summarised in the **Supplementary Table S5**. *ZNF185, TP63,* and *CDH1* expression data in normal or SCC samples were obtained from NCBI Gene Expression Omnibus. All the datasets with detailed description are summarised in the **Supplementary Table S4**. *ZNF185, TP63* and *CDH1* expression data and co-expression analysis in HNSCC samples from TCGA collection were performed using R2: Genomics Analysis and Visualization Platform (<http://r2.amc.nl/>). The expression values in different human tissues was extracted from GTEx portal (<https://www.gtexportal.org/>).

**Gene expression microarray**

Total RNA was extracted from HEKn knocked-down for ZNF185 and differentiated *in vitro* for 3 days. The gene expression microarray data with detailed experimental descriptions were deposited in the NCBI Gene Expression Omnibus database with accession number GSE102613.

**3D organotypic skin model experiments**

HEKn or Ker-CT cells (3x10^5^) were resuspended in CnT-PR growth medium (Cell-n-Tech, Bern, Switzerland) and seeded on MilliCell inserts (MilliPore, Burlington, Massachusetts, USA) into CnT-PR medium on 60-mm culture dishes. After 3 days, the medium was changed with CnT-3D Barrier medium (Cell-n-Tech). Sixteen hours later, the medium was removed and the inserts were put in low level CnT-3D Barrier medium to induce air-lifting. The medium was changed every 2 days for a total of 21 days. Inserts with organotypic tissue were fixed in 10% formalin buffered solution for 24 h and then processed and embedded in paraffin.

**Chromatin conformation capture (3C) assay**

The 3C assay was performed as described previously [56]. *In vitro* differentiated HEKn cells (1x10^7^) were used for the assay. Digestion of DNA was performed using BglII endonuclease (New England Biolabs, Ipswich, Massachusetts, USA). To detect the enhancer/promoter interaction, specific primers (3C-For and 3C-Rev, Supplementary Table 1) were used to amplify 152045666-152045589 and 152068082-152068157 regions on chromosome X, respectively (hg19). PCR products were resolved in 2% agarose gel, purified and sequenced. The 152062768-152062912 undigested region on chromosome X (hg19) was amplified with primers Un-For and Un-Rev (**Supplementary Table 1**) and used as a loading control.

**References**

Viticchiè G et al. p63 supports aerobic respiration through hexokinase II. Proc Natl Acad Sci U S A. 2015 Sep 15;112(37):11577-82.

Smirnov A et al. FOXM1 regulates proliferation, senescence and oxidative stress in keratinocytes and cancer cells. Aging (Albany NY). 2016 Jul;8(7):1384-97.

Sun BK et al. CALML5 is a ZNF750- and TINCR-induced protein that binds stratifin to regulate epidermal differentiation. Genes Dev. 2015 Nov 1;29(21):2225-30.

Kouwenhoven EN et al. Transcription factor p63 bookmarks and regulates dynamic enhancers during epidermal differentiation. EMBO Rep. 2015 Jul;16(7):863-78.
